# Nano-Se Assimilation and Action in Poultry and Other Monogastric Animals: Is Gut Microbiota an Answer?

**DOI:** 10.1186/s11671-017-2383-3

**Published:** 2017-12-04

**Authors:** Peter F. Surai, Ivan I. Kochish, Oksana A. Velichko

**Affiliations:** 1 Department of Microbiology and Biochemistry, Faculty of Veterinary Medicine, Trakia University, 6000 Stara Zagora, Bulgaria; 2 Department of Hygiene and Poultry Sciences, Moscow State Academy of Veterinary Medicine and Biotechnology named after K.I. Skryabin, Moscow, 109472 Russia; 3 Department of Animal Nutrition, Faculty of Agricultural and Environmental Sciences, Szent Istvan University, Gödöllo, H-2103 Hungary; 4 Department of Ecology and Genetics, Tyumen State University, Tyumen, 625003 Russia

## Abstract

Recently, a comprehensive review paper devoted to roles of nano-Se in livestock and fish nutrition has been published in the Nanoscale Research Letters. The authors described in great details an issue related to nano-Se production and its possible applications in animal industry and medicine. However, molecular mechanisms of nano-Se action were not described and the question of how nano-Se is converted into active selenoproteins is not resolved. It seems likely that the gut microbiota can convert nano-Se into selenite, H_2_Se or Se-phosphate with the following synthesis of selenoproteins. This possibility needs to be further studied in detail, and advantages and disadvantages of nano-Se as a source of Se in animal/poultry/fish nutrition await critical evaluations.

## Background

Recently, a comprehensive review paper devoted to roles of nano-Se in livestock and fish nutrition has been published in the Nanoscale Research Letters [1]. The authors described in great detail an issue related to nano-Se production and its possible applications in animal industry and medicine. Indeed, it is well established that many molecules presented as nanoparticles have unusual behaviour due to the new properties of such particles. In fact, in most of the published work relevant to animal nutrition, nanoparticles have size less than 100 nm. In such a state, the ultra-small size of the particles allows them to penetrate many biological barriers and be used as a delivery system for various elements including Ag, titanium, Se and others.

In this respect, Se is especially interesting, since it is proven that most areas worldwide are deficient in this element [2]. The deficiency in many cases is a human creation, since Se content in soils greatly varies, and what is more important, Se availability from soils is even more variable. In fact, soil acidification as a result of agricultural practices as well as usage of sulfur-containing fertilizers substantially decreased Se availability for plants and leads to low Se consumption by livestock, where grains (wheat, corn, barley) and oil seeds (soybean) comprise a major part of the diet. Therefore, Se supplementation of the commercial diets of poultry, farm animals and fish became a common practice and is used since the 1970s. Indeed, vitamin-mineral premixes are a major source of Se for the commercial production of eggs, meat/fish and milk.

What is relevant to the nano-Se story is the source of Se in the supplements. Indeed, the main supplemental form of Se is sodium selenite which is a by-product of copper production. With over more than 40 years of usage, sodium selenite has clearly demonstrated its advantages and disadvantages. In fact, due to the commercial feed Se supplementation, Se deficiency in livestock with clinical signs of deficiency practically disappeared. The exception is in ruminants, where sodium selenite can precipitate in acidic rumen environment, as well as due to technical limitations of the usage of feed supplements; Se deficiency is still seen worldwide and such applications as selenium injections and boluses are used. However, as it is rightly mentioned in the aforementioned review, Se is extremely important in commercially relevant stress conditions, especially for modern genetics of highly productive farm animals and poultry.

Today, the animal industry is moving from prevention of nutrient deficiencies to meeting the exact requirements of animals in important nutrients, including Se. Indeed, “precise nutrition” is a term describing this concept. In such conditions, it has become apparent that sodium selenite (as well as selenate) has a range of disadvantages as a feed supplement. First of all, it is quite a reactive compound and can be reduced in feed/premix to an unavailable metallic form by various nutrients, including ascorbic acid, and some feed ingredients. It can also be dissolved in feed moisture and converted into volatile compounds to be lost. Secondly, sodium selenite possesses pro-oxidant properties in a dose-dependent manner, which could have a negative effect on the animal/chick gut. Finally, selenium in the form of sodium selenite is poorly transferred to eggs, via placenta to foetuses, which is not able to build Se reserves in the body which can be used in stress conditions when Se requirement increases but feed consumption usually decreases. Sodium selenite is also toxic for animals and humans in excess. However, we do not agree with a conclusion in the aforementioned review [1] that Se toxicity is a problem for the animal industry. It is only related to human error in calculation or in mixing feeds. The safe window for selenium is quite narrow (the usual dose of Se supplementation for poultry and pigs is about 0.3 ppm, while a negative effect would be observed at doses of sodium selenite exceeding 1–2 ppm), but modern feed mill equipment gives an opportunity of good feed mixing avoiding problems of toxicity.

Advances in analytical sciences were a driving force in discovery that the main form of Se in major feed ingredients is SeMet, comprising more than 50% of total Se in corn, soybean, wheat, barley, etc. [2]. Therefore, during evolution, the digestive system of animals was adapted to this form of selenium, and as a result, SeMet is more efficiently assimilated from the diet and non-specific incorporation into body proteins builds Se reserves. Indeed, a range of organic Se sources appeared in the market including Se-Yeast, pure SeMet and OH-SeMet (2-hydroxy-4-(methylthio)butanoic acid-Se), with OH-SeMet to show maximum efficacy.

The main lack of knowledge in the nano-Se issue is related to its metabolism and particularly its conversion to H_2_Se with the following SeCys synthesis and incorporation into selenoproteins. In the review [1], only very few selenoproteins, including glutathione peroxidase, are mentioned, while at least 25 selenoproteins are identified in humans and animals. It is generally accepted that the major role of Se in human/animal nutrition is related to the synthesis of selenoproteins possessing unique catalytic properties and more than half of them are involved in redox balance maintenance and antioxidant defences [3]. In the aforementioned review [1], the direct antioxidant properties (reduction of ROS) of nano-Se are mentioned as a possible mechanism of its action. However, Se concentration in major animal/poultry tissues including the liver and muscles usually does not exceed 800–900 ng/g fresh tissue which is in the range of 10 μM, and in plasma, the Se level is about 0.2–0.3 μg/ml or 2–3 μM, while in the most cited work devoted to antioxidant properties of nano-Se in vitro, the Se concentrations were tested and showed antioxidant effects 5–10-folds higher [4]. Furthermore, for an antioxidant compound to be an effective free-radical scavenger, it is important to have the right concentration of the antioxidant in the right place at the right time. This would complicate the issue further, and therefore, it seems unlikely that nano-Se could have a direct AO effect in the biological systems.

Therefore, similar to other forms of selenium used in animal diets, an antioxidant effect of nano-Se is related to selenoprotein gene expression and protein synthesis. Indeed, in the review [1], there are several references confirming a positive effect of nano-Se on the GSH-Px activity. Now the question is *how* nano-Se is converted into active selenoproteins. In the review [1], there is a suggestion (without a reference) that nano-Se can be converted to selenophosphate with the following Se-protein synthesis. This suggestion should be experimentally proven. The second suggestion that nano-Se can be converted to SeMet is fundamentally wrong, since SeMet cannot be synthesized in the human/animal body; only plants and bacteria can produce it [5].

There are very attractive suggestions that gut microbiota can oxidize nano-Se into selenite/selenate or reduce it into H_2_Se with the following synthesis of selenoproteins [6, 7]. Recently, some experimental evidence have been provided to prove that nano-Se particles can be dissolved and oxidized to inorganic oxoanions of Se in the gut in the presence of microbiota before their absorption [6]. Furthermore, a hypothetical schematic diagram showing an intracellular dynamic cycle of endogenous selenium nanoparticles (SeNPs) was proposed [7]. In fact, it was suggested that elemental selenium could be re-oxidized by superoxide radicals into selenite. There is also a possibility that gut microbiota can perform/accelerate this process. Indeed, four major biological transformations of Se are proven to occur in nature including reduction, oxidation, methylation, and demethylation [8]. Data accumulated for the last three decades clearly indicate that microorganisms play a major role in the selenium cycle in the environment by participating in both oxidation and reduction reactions [9]. Interestingly, the microbial oxidation of Se^0^ to Se^4+^ by a group of unidentified autotrophic bacteria was discovered more than 90 years ago [10]. Furthermore, oxidation of elemental selenium to selenite by a heterotrophic bacterium, *Bacillus megaterium*, isolated from soil was reported much later [11]. Indeed, Se^0^ oxidation in soils was shown to occur at a relatively slow rate and to be largely biotic in nature and yields both SeO_3_^2−^ and SeO_4_^2−^ [12]. Furthermore, the microbial oxidation of elemental selenium (Se^0^) by chemoheterotrophs and chemoautotrophic thiobacilli was confirmed by using ^75^Se^0^ as a tracer [13]. The authors showed that soil slurries were able to oxidize Se^0^ with SeO_3_^2−^ and SeO_4_^2−^ formation. Interestingly, microbial inactivation in soil by autoclaving or chemical treatments inhibited the process. Furthermore, cultures of sulfur-oxidizing bacterium *Thiobacillus* ASN-1 are shown to perform the oxidation of Se(0) with enzymes that are used for generating energy from reduced sulfur compounds [13]. Furthermore, heterotrophic bacterium which can oxidize Mn(II) or Fe(II) (*Leptothrix* MnB1) was shown to oxidize Se^0^ with the formation of SeO_3_^2−^ as the major product of reaction. Interestingly, the reaction was shown to depend on an electron donor such as acetate or glucose [13]. The oxidation of Se^0^ by various bacteria has not been fully addressed and is an area of important research opportunities. In fact, a great variety of microbes residing in the gut provides necessary conditions for various Se conversions. For example, when nano-Se particles were incubated with lactic acid bacteria (*Lactobacillus delbrueckii* subsp*. bulgaricus* LB-12), organic Se compounds (mainly SeCys and SeMet) were produced and also nano-Se particles were partially dissolved and non-metabolically transformed into inorganic selenium, probably with the assistance of substances excreted by the bacteria cell wall [14].

Alternatively, elemental selenium can be reduced/converted into selenide by a selenite-respiring bacterium (for example, *Bacillus selenitireducens*). The aforementioned reaction conducted by the bacteria with the incomplete oxidation of the electron donor lactate to acetate as follows was presented as follows [7]:

$$ {\mathrm{C}}_2{\mathrm{H}}_4{\mathrm{OHCOO}}^{-}+2{\mathrm{Se}}^0+2{\mathrm{H}}_2\mathrm{O}\to {\mathrm{C}\mathrm{H}}_3{\mathrm{C}\mathrm{OO}}^{-}+2{\mathrm{H}\mathrm{Se}}^{-}+{{\mathrm{H}\mathrm{CO}}_3}^{-}+3{\mathrm{H}}^{+} $$

**Fig. 1:**
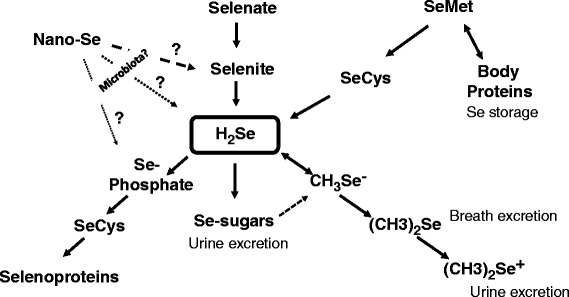
Schematic model showing metabolic conversions of various forms of Se in animals. It is suggested that gut microbiota could convert nano-Se into selenite, H_2_Se or Se-phosphate with the following SeCys synthesis and incorporation into selenoproteins

Therefore, the free energies for the reaction (∆G^I^) is shown to be − 2.8 kcal/mol e^−^. This shows that in *Bacillus selenitireducens*, the reduction mechanism involves energy conservation by using Se-specific dissimilatory enzymes [15]. Indeed, a selenite-respiring bacterium, *Bacillus selenitireducens*, could produce significant levels of Se^− 2^ (as aqueous HSe−) using Se^0^ as a substrate [16]. Earlier it was demonstrated that red selenium was reduced by obligate acidophile *Thiobacillus ferrooxidans* under acidic (pH 3), anaerobic conditions with H_2_Se production at a rate of 0.03 μmol/mg protein/h [17]. Furthermore, another anaerobic bacterium *Veillonella atypica* was shown to be able to reduce selenium oxyanions to form elemental selenium with its further reduction by the bacterium to form reactive selenide [18]. Interestingly, more than 45 years earlier, it was described that extracts of Micrococcus lactilyticus (*Veillonella alcalescens*) were able to quantitatively reduce colloidal selenium into H_2_Se [19]. Recently, genes encoding YedE and YedF proteins have been considered as new candidate genes involved in Se metabolism in prokaryotes including bacteria [20]. Indeed, both YedE, a predicted Se transporter, and YedF, a redox protein, could be involved in the metabolic transformation of selenium in bacterial cells. Therefore, the principal ability of various microbes to oxidize or reduce elemental selenium was proven (Table 1); however, there is a need for further investigations to answer a question if such reactions take place in the animal gut. Interestingly, from all bacterial species mentioned in Table 1, anaerobic, gram-negative bacteria of the genus *Veillonella* deserve special attention*.* Indeed, *Veillonellae* are found in the alimentary canal of warm-blooded animals [21]. In fact, in food animals, *Veillonella* strains are detected regularly as indigenous inhabitants of all sections of the gastrointestinal tract ([22] and references there), including the upper gastrointestinal tract [23] and caeca [24] of chickens. Indeed, an additional research is needed to elucidate the fate and mechanisms of the possible conversions of nano-Se in the gastrointestinal tract of animals. The nano-Se metabolism depends on the nanoparticle composition, including the nanoparticle coating agent. A proposed scheme of nano-Se participation in selenoprotein synthesis is shown in Fig. 1, and a basic understanding of the nano-Se metabolism including absorption, distribution, and clearance is of great importance in animal/poultry sciences [25].

**Table 1:** Metallic selenium transformation by microorganisms

Se conversion	Organism used	Reference
Se^0^→SeO_3_^2−^	*Bacillus megaterium*	[11]
Se^0^→SeO_3_^2−^; SeO_4_^2−^	*Thiobacillus ASN-1, Leptothrix MnB1*	[13]
Se^0^→Se^2−^	*Thiobacillus ferrooxidans*	[17]
Se^0^→Se^2−^	*Bacillus selenitireducens*	[15, 16]
Se^0^→Se^2−^	*Veillonella alcalescens*	[19]
Se^0^→Se^2−^	*Veillonella atypica*	[18]

The mammalian/avian gastrointestinal tract harbours trillions of commensal microorganisms, collectively known as the microbiota [26]. For example, chicken intestinal tract is composed of duodenum, jejunum, ileum, cecum, and colon, and there are significant differences in microbiota concentration and composition between the aforementioned gut sections [27]. Interestingly, the cecum is characterized by the most complex microbial community dominated by the phyla *Firmicutes*, *Bacteroidetes*, *Actinobacteria* and *Proteobacteria* [28]. On the other hand, at the genus level, the major microbial genera across all gut sections were shown to be *Lactobacillus*, *Enterococcus*, *Bacteroides*, and *Corynebacterium* [27]. Furthermore, *Bacteroides* was shown to be the dominant group in the cecum, while *Lactobacillus* was predominant in the small intestine sections (duodenum, jejunum and ileum; [27]). In this complex intestinal ecosystem, there could be a range of microbes able to facilitate oxidation/reduction of nano-Se particles, and this assumption awaits further investigation.

From one side, some microbes would use Se for their own needs to synthesize microbial selenoproteins and compete with the host for this element. It is well known that Se is an important element for a variety of organisms in almost all bacterial phyla; however, it seems likely that only one third of characterized bacteria use this element in their metabolism [29]. Indeed, in Se deficiency, there is a competition between bacteria and the host for the available selenium, and germ-free animals have a lower selenium requirement than conventionally colonized animals [30]. Recent results indicate that dietary Se supplementation can affect both the composition and diversity of the existing microbiota and establishment of gastrointestinal microflora [31]. For example, weaned beef calves fed selenium-enriched alfalfa hay were shown to have an enriched nasal microbiota compared with control animals [32]. Possible effects of Se in various forms and concentrations on gut microbiota await further investigation.

On the other hand, it seems likely that there is an active uptake of Se by microbiota, and this process depends on the Se form used. In fact, the uptake of SeMet by colon microbiota was shown to be much more efficient compared to the uptake of selenate [33]. However, whether bacteria composition and concentration affect the absorption of selenium compounds in the gut has not been investigated. It is generally accepted that the gut microbiota is responsible for the excretion of excess selenium by its methylation and elemental Se formation [34]. Interestingly, in rats fed SeMet, this form of Se was found in all segments of the gut. However, the level of Se in the ileum, cecum and colon were significantly higher than that in the corresponding sections of rats after probiotic treatment [34]. This could mean that *Streptococcus Salivarius*, *Lactobacillus rhamnosus*, *Lactobacillus acidophilus* and *Bifidobacterium lactis* were delivered to the gut with probiotic effected Se metabolism mainly in the distant gut. Indeed, the effect of different bacteria in the conversion of Se should be identified to understand the roles of each segment of the gut in Se metabolism and assimilation. Furthermore, reflux of the fluid from the upper part from the large intestine back to the small intestine may be responsible for the absorption of H_2_Se and other forms of Se from the intestine. In fact, recently, it has been shown that chickens are characterized by reverse peristaltic contractions which could move the marker from the cloaca to the gizzard [35].

Direct involvement of nano-Se into selenoprotein synthesis could be expected, since in cell culture nano-Se increased selenoproteins (GSH-Px and TR activity). However, the recent understanding of the selenoprotein expression priority could complicate this issue. In fact, many selenoproteins are oxidative stress-regulated. In particular, GSH-Px1, GSH-Px4 and TR1 were shown to be upregulated in response to oxidative stress [36], and such a response was more pronounced when Se supply was limiting. It should be mentioned that the aforementioned response depends also on the level of oxidative stress, because it is true in mild oxidative stress, but at an extremely high level, some other mechanisms are activated [2].

It could well be that in cell culture, increased expression/activities of such selenoproteins are a response to an oxidative stress created by nano-Se, but not a reflection of improved Se supply. Therefore, there should be some caution in interpretation of the results based on cell-culture studies. An additional confirmation of the stress-related changes in biological systems due to nano-Se supplementation came from the recent study showing that biogenic nano-Se could activate the nuclear factor (erythroid-derived-2)-like 2 (Nrf2) and increase the expression of its downstream genes, responsible for antioxidant synthesis in dose- and time-dependent manners [37]. Furthermore, the authors found that the knockdown of Nrf2 significantly blocked the antioxidative effect of such nano-Se particles.

When critically analysing positive effects of nano-Se supplementation on productive and reproductive performance of poultry, farm animals and fish, it is necessary to take into account that in most cases, Se did not improve the performance but rather prevented performance deterioration due to environmental or nutritional constraints. In many cases, animal experiments were conducted at extremely low Se background level, and therefore, adding Se in any available form can have positive effects.

The challenges of nano-Se commercialization to be used as a feed additive could be as follows:

1. It is necessary to understand molecular mechanisms of nano-Se absorption, assimilation and action at the cellular, subcellular and gene levels. Without such data, it would be difficult to have reproducible results and find proper explanations of the observed effects. For example, in a recently published paper, only few genes (18 proteins and none of them are directly related to Se metabolism) were affected in the liver due to overdose of nano-Se in chickens [38], while it is known that other forms of dietary Se, including SeMet, can affect few hundred genes.
2. It is important to understand if nano-Se can build any Se reserves in the body, like SeMet, and if those reserves are available in stress conditions.
3. It is necessary to design a technology able to provide nano-Se particles of the same size, stability and reasonably good (at least 6–12 months) storability [2].
4. It is likely that microbial probiotics can be useful in the conversion of nano-selenium used as feed additive. From the one hand, such probiotics can contain specific microorganisms helping nano-Se assimilation in the gut (e.g. *Veillonella* species). On the other hand, probiotics enriched with Se could be another possibility [34, 39]
5. Side effects, risks and environmental concerns should be addressed in full. Indeed, further research is required in order to inform policy makers and regulatory bodies about the nanotoxicological potential of nano-Se [40]. In particular, a very small size of particles in dry form makes the product very dusty, and unusual nano-Se particle behaviour, once in the body, gives a warning with the main concern for feed mill workers’ protection. Since the gut microbiota is responsible for the excretion of excess selenium by its methylation and elemental Se formation [34], the enhancement/modulation of the microbiota could open a new horizon to deal with possible nano-Se toxicity.
6. Positive effects of nano-Se in animal nutrition should not overshadow possible detrimental consequences of its usage. Indeed, nanoparticle behaviour in various conditions could differ substantially, and before we understand how to control that behaviour, nano-Se usage on a wide industrial scale should not be possible. For example, when considering nano-Se absorption, it is necessary to mention the so-called Trojan horse effect, when nanoparticles may have permeation-enhancing properties for other substances in the gut [41]. This could create some problems, since there is a range of “unwanted” compounds in the feed and the gut is protective against their absorption. Indeed, nano-Se behaviour in the gut warrants further investigation.
7. In the aforementioned review [1], substantial attention is given to antimicrobial and anti-cancer properties of nano-Se, and it seems likely that the unique properties of nanoparticles could help in fighting various disease conditions. Indeed, nano-Se can be considered as a new drug to be used in various medical conditions, including cancer therapy, while its usage as an effective feed additive is rather questionable. Future research has to answer those important questions and concerns.

## Conclusion

For the last few years, a range of papers were published devoted to nano-Se and the topic is quickly developing. However, before this form of Se can find a way to commercial poultry/animal production, it is necessary to understand and explain how nano-Se is converted into active selenoproteins. One of the possible mechanisms/pathways of nano-Se action could be mediated by the gut microbiota which could convert nano-Se into selenite, H_2_Se or Se-phosphate with the following synthesis of selenoproteins (Fig. 1). There is some evidence that in nature, bacteria could reduce or oxidize metallic Se (Se^0^) with the production of Se^−2^ or Se^+4^ and Se^+6^ respectively. Among microorganisms involved in Se redox changes, the genus *Veillonella* deserves special attention because of the presence of such bacteria in the gut of food animals, including chicken. The possibility of participation of gut microbiota in nano-Se assimilation and metabolism needs to be further investigated in detail, and advantages and disadvantages of nano-Se as a source of Se in animal/poultry nutrition await further critical evaluations.
